# MicroRNA expression in HTLV-1 infection and pathogenesis

**DOI:** 10.1186/1742-4690-8-S1-A156

**Published:** 2011-06-06

**Authors:** Donna M D'Agostino, Katia Ruggero, Marta Biasiolo, Stefania Bortoluzzi, Cynthia A Pise-Masison, Alberto Corradin, Katia Basso, Alessandro Guffanti, Gianluca De Bellis, Giorgio Corti, Paola Zanovello, Vincenzo Bronte, Vincenzo Ciminale

**Affiliations:** 1 Department of Oncology and Surgical Sciences, University of Padova, Padova, Italy; 2 Istituto Oncologico Veneto-IRCCS, Padova, Italy; 3 Department of Biology, University of Padova, Padova, Italy; 4 Animal Models and Retroviral Vaccines Section, NCI, NIH, Bethesda, MD, USA; 5 Department of Information Engineering, University of Padova, Padova, Italy; 6 Institute for Cancer Genetics, Columbia University, New York, USA; 7 Institute of Biomedical Technologies, National Research Council, Milan, Italy

## 

Our laboratory is examining the profiles of microRNA expression in ATLL cells and infected T-cell lines using microarrays and small RNA libraries.

Microarray analysis of ATLL samples revealed 6 upregulated and 21 downregulated microRNAs in ATLL cells compared to CD4+ T-cell controls. Potential targets for deregulated microRNAs were identified by integrating microRNA and mRNA expression profiles. Current experiments are aimed at verifying these predicted microRNA-target interactions.

Mass sequencing of small RNA libraries prepared from normal CD4+ cells and two chronically infected T-cell lines yielded panels of known and candidate new microRNAs for each library. Comparison of frequencies of known microRNAs led to the identification of a small number of microRNAs differentially expressed in both infected cell lines compared to controls. Most of the candidate new microRNAs were intragenic with poor species conservation, suggesting that they might have particular roles in human T-cell function. Two sequences mapped to the HTLV-1 genome, suggesting that the virus may produce its own microRNAs. Further analyses of the new cellular and viral microRNA candidates are in progress.

